# Genetically-determined systemic lupus erythematosus requires persistent CAR-T therapy: challenging the transient depletion paradigm

**DOI:** 10.1093/jmcb/mjag002

**Published:** 2026-02-09

**Authors:** Baichuan Liu, Wenchen Ruan, Jingwei Jiang, Jinjiao Wang, Li Li, Xindong Liu, Haopeng Wang

**Affiliations:** School of Life Science and Technology, ShanghaiTech University, Shanghai 201210, China; Lingang Laboratory, Shanghai 200031, China; Institute of Pathology and Southwest Cancer Center, Southwest Hospital, Third Military Medical University, Chongqing 400038, China; School of Life Science and Technology, ShanghaiTech University, Shanghai 201210, China; School of Life Science and Technology, ShanghaiTech University, Shanghai 201210, China; School of Life Science and Technology, ShanghaiTech University, Shanghai 201210, China; Institute of Pathology and Southwest Cancer Center, Southwest Hospital, Third Military Medical University, Chongqing 400038, China; Jinfeng Laboratory, Chongqing 401329, China; School of Life Science and Technology, ShanghaiTech University, Shanghai 201210, China; Changping Laboratory, Beijing 102206, China

**Dear Editor**,

Chimeric antigen receptor (CAR)-T cell therapy, initially developed as a curative treatment for B-cell hematologic malignancies, has recently demonstrated transformative potential in autoimmune diseases, including systemic lupus erythematosus (SLE), systemic sclerosis, inflammatory bowel disease, and multiple sclerosis (Mougiakakos et al., 2021; Müller et al., 2024). Of particular note, SLE treatment shows emerging evidence of durable drug-free remissions.

The therapeutic paradigm for CAR-T cell therapy in SLE fundamentally differs from oncologic applications through a distinctive ‘deplete-and-reset’ mechanism. Emerging clinical evidence increasingly supports the concept that transient anti-CD19 CAR-T cell therapy achieves curative outcomes in SLE by inducing deep B cell depletion that eliminates pathogenic autoreactive clones and long-lived autoantibody-producing plasma cells. Subsequently, immune reconstitution generates a renewed, naive B cell repertoire devoid of SLE-associated autoreactivity. This immunological reset, facilitated by the transient CAR-T cell therapy, establishes immune tolerance and enables sustained drug-free remission, fundamentally contrasting with cancer therapeutics where prolonged CAR-T cell persistence is essential for tumor control.

However, the multifactorial pathogenesis of autoimmune diseases may fundamentally challenge the curative potential of transient CAR-T therapy. SLE exemplifies this complexity, arising from genetic predisposition, human leukocyte antigen polymorphisms, and environmental triggers (e.g. ultraviolet exposure and viral infection). Notably, monogenic mutations directly causing human SLE have been identified, with DNASE1L3 deficiency serving as a paradigmatic example. DNASE1L3 loss-of-function mutations have been conclusively demonstrated to cause familial, early-onset SLE in humans through impaired clearance of extracellular DNA (Al-Mayouf et al., 2011). This results in pathological accumulation of extracellular double-stranded DNA (dsDNA), creating continuous antigenic stimulation and driving chronic autoimmune inflammation that manifests as classical SLE phenotypes in affected patients. We hypothesize that, in these genetically determined cases, transient CAR-T therapy faces a critical challenge: while initial B cell depletion may provide temporary relief, immune reconstitution will inevitably regenerate autoreactive B cells in the presence of the persistent dsDNA stimulus, leading to disease relapse and thus requiring sustained therapeutic intervention.

To test this hypothesis, we developed a controllable CAR-T system enabling precise modulation of therapeutic persistence to model transient vs. persistent B cell depletion (Figure 1A). Since conventional mouse CAR-T cells often persist for extended periods *in vivo* (Kansal et al., 2019; Jin et al., 2021), we engineered CAR-T cells co-expressing THY1.1 with a murine CD19 (mCD19) CAR via a P2A self-cleaving peptide (Szymczak et al., 2004). This design permits selective depletion of CAR-T cells *in vivo* through antibody-dependent cellular cytotoxicity, thereby enabling controlled B cell reconstitution that mirrors clinical observations. Flow cytometric analysis confirmed robust surface expression of both THY1.1 and the CAR on transduced murine T cells (Figure 1B), with preserved effective cytotoxic functionality against CD19^+^ target cells (Figure 1C).

**Figure 1:**
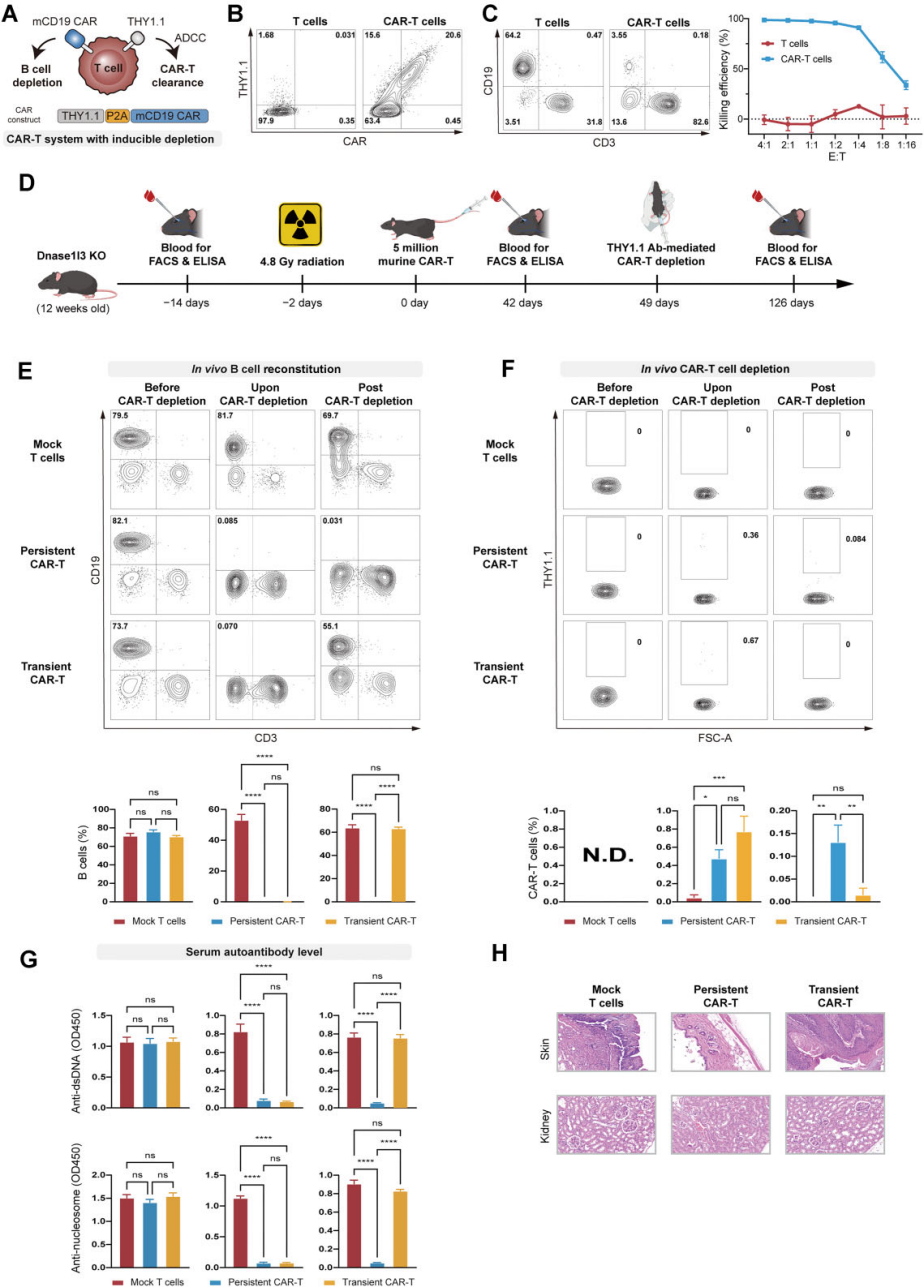
Development of a controllable CAR-T system for studying CAR-T persistence in treating SLE. (**A**) Schematic representation of the controllable CAR-T construct. THY1.1 was linked to mCD19 CAR via a P2A self-cleaving peptide. (**B**) Flow cytometric analysis showing surface expression of THY1.1 and CAR on mouse T cells. (**C**) Cytotoxic activity of mouse CAR-T cells against B cells at different effector-to-target (E:T) ratios over 18 h. (**D**) Experimental timeline of the Dnase1l3 KO mouse study. Mice were divided into three groups: mock, persistent CAR-T, and transient CAR-T. Both CAR-T groups were infused with 5 × 10⁶ gender-matched CD8⁺ mouse CAR-T cells 2 days after irradiation, while the mock group received an equal number of untransduced CD8⁺ mouse T cells. On Day 49, mice in the transient CAR-T group were intraperitoneally injected with anti-THY1.1 antibody to eliminate CAR-T cells. (**E**) Flow cytometry plots showing the proportion of B cells within lymphocytes before and after CAR-T infusion. A representative image from each group at each time point is shown; detailed data are provided in Supplementary Figure S1. (**F**) Flow cytometry plots showing the proportion of CAR-T cells within T cells before and after CAR-T infusion. A representative image from each group at each time point is shown; detailed data are provided in Supplemenary Figure S2. (**G**) Serum levels of anti-dsDNA and anti-nucleosome antibodies were measured by ELISA before and after CAR-T infusion. Serum samples were diluted 1:6. (**H**) Representative images of hematoxylin and eosin-stained skin and kidney tissue sections from Dnase1l3 KO mice at Week 18, comparing tissue morphology and cellular architecture among groups. Bar graphs represent mean ± SEM. N.D., not detected. Statistical differences among groups at designated time points were analyzed by one-way analysis of variance (ANOVA) (ns, not significant; *P < 0.05, **P < 0.01, ***P < 0.001, and ****P < 0.0001).

We employed the *Dnase1l3*⁻/⁻ (Dnase1l3 KO) mouse model to recapitulate human SLE pathogenesis, as previously reported (Sisirak et al., 2016). The experimental workflow was described in Figure 1D. Baseline assessment performed 2 weeks prior to CAR-T infusion revealed normal B cell frequencies but elevated serum anti-dsDNA and anti-nucleosome antibodies in all groups (Figure 1E and F), confirming SLE pathology. The mock control group maintained these elevated autoantibody levels throughout the study, serving as disease controls.

Six weeks post-CAR-T administration, CAR-T-treated groups showed complete B cell elimination, with consequently undetectable autoantibody levels (Figure 1E and G), consistent with clinical observations. In the persistent CAR-T group, mice maintained B cell depletion and autoantibody suppression throughout the observation period (Figure 1G). In contrast, in the transient CAR-T group, anti-THY1.1 antibody injection resulted in CAR-T depletion (Figure 1F) and consequently led to B cell reconstitution by Week 18. Importantly, this B cell recovery was accompanied by recurrent anti-dsDNA and anti-nucleosome antibody production, with consistent trends observed in the pathological sections of the skin and kidney (Figure 1E–H), demonstrating disease relapse upon loss of CAR-T-mediated B cell control.

These results challenge the prevailing assumption that transient B cell depletion is universally sufficient for autoimmune disease control. Our findings demonstrate that genetically determined SLE, particularly caused by DNASE1L3 deficiency, is prone to relapse following initial B cell depletion induced by transient CAR-T therapy, whereas persistent CAR-T therapy achieves sustained remission. The observed disease recurrence following transient B cell elimination suggests that reconstituting B cells may continuously encounter persistent pathogenic DNA antigens, potentially regenerating autoreactive responses. These findings indicate that, for such SLE cases, persistent CAR-T therapy may be essential rather than optional to achieve sustained remission, revealing that genetic context critically influences therapeutic outcomes.

Given these findings, we recommend pre-therapeutic genetic screening for autoimmune patients considered for CAR-T therapy to identify high-risk individuals harboring mutations in DNASE1L3 or functionally related genes and tailor appropriate treatment strategies. Notably, the conceptual framework revealed by our study is likely applicable beyond DNASE1L3 deficiency to other genetically defined human autoimmune disease contexts characterized by persistent defects in immune tolerance, impaired clearance of self-antigens, or enhanced survival of autoreactive B cells (Supplementary Table S1). Such contexts include pathogenic mutations affecting nucleic acid clearance pathways (e.g. TREX1), loss-of-function variants in inhibitory B cell signaling molecules (such as FCGR2B), and genetic variants associated with increased B cell survival signaling (such as BAFF). In these settings, the continuous generation and survival of autoreactive B cell populations may predispose newly reconstituted B cells to reacquire pathogenic phenotypes following depletion, thereby increasing the likelihood of disease recurrence and suggesting a potential need for sustained, rather than transient, therapeutic suppression.

Currently, the pathogenic mechanisms underlying B cell-mediated autoimmune diseases remain incompletely understood, and the associations between specific genetic variants and disease onset require further elucidation. Importantly, conducting genetic analyses in autoimmune patients who relapse after CAR-T therapy may help refine and expand the catalog of high-risk genes associated with disease recurrence.

From a therapeutic perspective, achieving sustained B cell suppression in high-risk patients, as required by the continuous antigenic stimulation demonstrated in our study, can be accomplished through persistent CAR-T cells that maintain durable B cell control through long-term cellular persistence, though this necessitates careful monitoring for safety concerns including infection risk. Multiple strategies are currently being explored to enhance CAR-T persistence, including engineering CAR-T cells to express supportive cytokines (Castelli et al., 2026; Jiang et al., 2026; Zuo et al., 2026), optimizing intracellular signaling domains to reduce exhaustion and promote memory differentiation (Li et al., 2020), refining CAR tonic signaling (Chen et al., 2023), and employing pharmacologic approaches to induce intermittent ‘rest’, which has been shown to alleviate or reverse T cell exhaustion (Weber et al., 2021). Alternatively, whether transient modalities such as bispecific antibodies, CAR-NK cells, or LNP-based *in vivo* CAR-T therapies could achieve comparable sustained suppression through sufficiently frequent periodic administration remains to be clinically validated.

*[Supplementary material is available at Journal of Molecular Cell Biology online. We thank Dr Chenqi Xu (Center for Excellence in Molecular Cell Science, Chinese Academy of Sciences) for providing the mCD19 CAR plasmid. We also thank the staff members of the Animal Core Facility at ShanghaiTech University for their support in mouse housing and care, as well as the staff of the institutional technical platforms, including the Flow Cytometry and Molecular Biology Platforms, for their technical support. This work was supported by grants from the National Science Fund for Distinguished Young Scholars (82525032), Changping Laboratory, the Program of Shanghai Academic Research Leader, the Central Guidance on Local Science and Technology Development Fund (YDZX20233100001002), Shanghai Municipal Health Commission Collaborative Innovation Cluster Project (2024CXJQ02), and Shanghai Science and Technology Commission (24J22800700).]*

## Supplementary Material

mjag002_Supplemental_File
