# Supplementary material for: Genetically-determined systemic lupus erythematosus requires persistent CAR-T therapy: challenging the transient depletion paradigm
Source: J Mol Cell Biol. 2026 Feb 9;18:mjag002. doi: 10.1093/jmcb/mjag002 (PMC13344418; doi:10.1093/jmcb/mjag002)
Supplement: mjag002_Supplemental_File [file mjag002_supplemental_file.pdf]

Supplementary material

Supplementary Figures

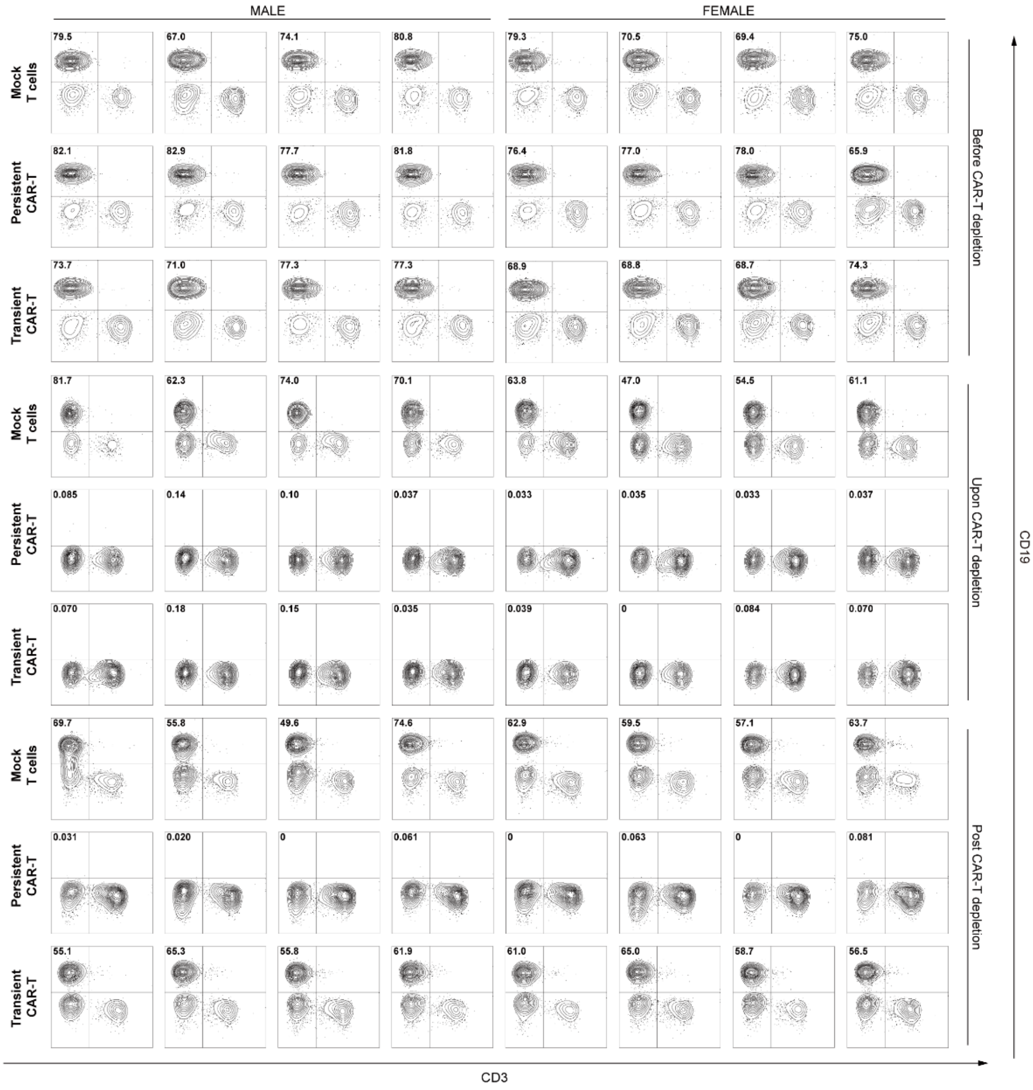

Supplementary Figure S1 Flow cytometry plots showing the detailed proportion of B cells within lymphocytes at each time point before and after CAR-T infusion.

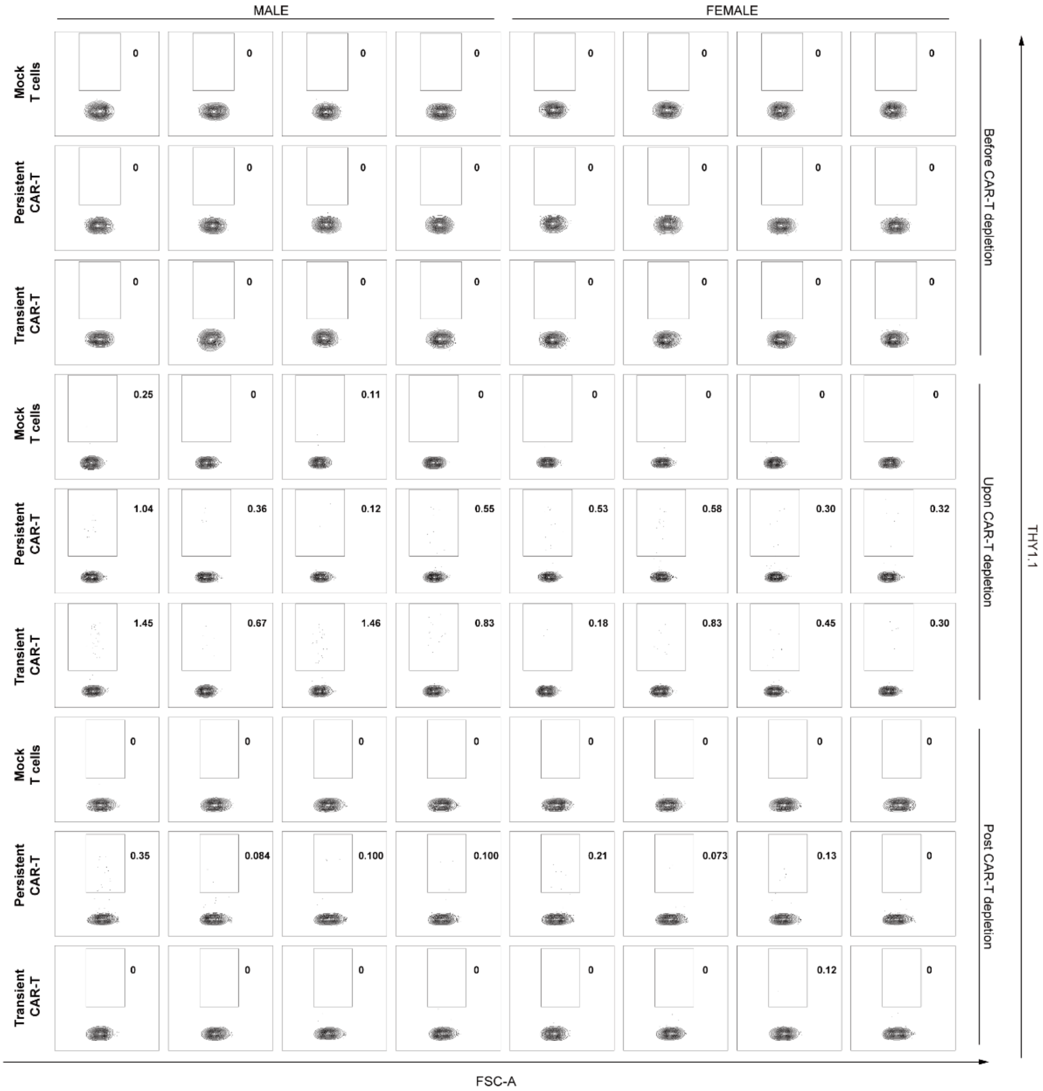

Supplementary Figure S2 Flow cytometry plots showing the detailed proportion of CAR-T cells within T cells at each time point before and after CAR-T infusion.

Supplementary Table

Supplementary Table S1. Genetic and pathogenic contexts in which persistent CAR-T therapy may be required in B cell-mediated autoimmune diseases.

Disease Type	Gene Mutation	Pathogenic Mechanism
Systemic lupus erythematosus (SLE)	TREX1 loss-of-function mutations	Accumulation of cytosolic DNA → chronic type I interferon signaling → expansion and activation of autoreactive B cells
	TLR7 gene duplication or gain-of-function (X-linked)	Enhanced RNA sensing → amplified BCR–TLR7 dual signaling → excessive activation of autoreactive B cells
	UNC93B1 pathogenic variants	Dysregulated endosomal trafficking of TLR7/9 → exaggerated nucleic acid–driven B cell activation
	FCGR2B loss-of-function or reduced expression	Loss of inhibitory FcγRIIB signaling → failure of immune complex–mediated negative feedback → sustained autoantibody production
	PTPN22 R620W variant	Impaired negative regulation of BCR signaling → defective central and peripheral B cell tolerance
	BANK1 risk variants	Enhanced BCR signaling scaffold function → increased calcium mobilization → promotion of autoantibody production
	BLK risk variants	Augmented proximal BCR signaling → reduced tolerance threshold during B cell development
	IRF5 / IRF7 gain-of-function variants	Amplified type I interferon responses → potentiation of TLR-mediated B cell activation and class-switch recombination
Rheumatoid arthritis (RA)	PTPN22 R620W variant	Dysregulated BCR signaling → survival of autoreactive B cells → production of anti-citrullinated protein antibodies
	STAT4 risk variants	Enhanced Tfh/Th1 differentiation → excessive B cell help → pathogenic autoantibody generation
Primary Sjögren’s syndrome (pSS)	TNFSF13B (BAFF) overexpression–associated variants	Increased B cell survival signals → failure to eliminate autoreactive B cell clones
	PRDM1 (BLIMP-1) pathogenic variants	Dysregulated plasma cell differentiation → accumulation of long-lived autoantibody-secreting cells
Autoimmune hemolytic anemia (AIHA)	FCGR2B functional deficiency	Unrestrained production of anti–red blood cell antibodies → immune-mediated hemolysis
Immune thrombocytopenia (ITP)	FCGR2B loss-of-function variants	Impaired inhibitory signaling → persistent anti-platelet antibody production
Autoimmune lymphoproliferative–like disease	CTLA4 haploinsufficiency	Loss of Tfh regulation → excessive B cell help → broad autoantibody responses
Multisystem autoimmunity	DEF6 or FERD3 pathogenic variants	Dysregulated Tfh–B cell axis → uncontrolled germinal center reactions

This table summarizes representative human B cell-mediated autoimmune diseases, their causative or high-risk genetic mutations identified in patients, and the corresponding B cell-centric pathogenic mechanisms. These genetic alterations converge on fundamental processes, including failure of central or peripheral B cell tolerance, defective clearance of self-nucleic acids, exaggerated nucleic acid-sensing pathways, and loss of inhibitory immune checkpoints, thereby enabling the continuous generation and survival of autoreactive B cell clones and sustained autoantibody production. In such genetically driven disease contexts, transient B cell depletion is unlikely to provide durable remission, as newly generated B cells are predisposed to reacquire pathogenic phenotypes. Therefore, these conditions are predicted to require sustained therapeutic pressure, such as persistent CAR-T cell activity, to maintain long-term disease control and prevent relapse.
